# Consequences of Exposure to Hypobaric Hypoxia Associated with High Altitude on Spermatogenesis and Seminal Parameters: A Literature Review

**DOI:** 10.3390/cells13070592

**Published:** 2024-03-29

**Authors:** Carlos Cornejo-Guerra, Camila Salazar-Ardiles, Patricio Morales, David C. Andrade

**Affiliations:** 1 Exercise Applied Physiology Laboratory, Centro de Investigación en Fisiología y Medicina de Altura (FIMEDALT), Departamento Biomédico, Facultad de Ciencias de la Salud, Universidad de Antofagasta, Antofagasta 1271155, Chile; carloscornejo75@gmail.com (C.C.-G.); nickolsalazar@gmail.com (C.S.-A.); 2 Laboratorio de Biología de la Reproducción, Departamento Biomédico, Facultad de Ciencias de la Salud, Universidad de Antofagasta, Antofagasta 1271155, Chile; patricio.morales@uantof.cl

**Keywords:** hypoxia, male infertility, spermatogenesis

## Abstract

Preclinical research has provided compelling evidence indicating that exposure to hypobaric hypoxia (HH) results in a deterioration of spermatogenesis. This adverse effect extends to the underlying molecular mechanisms, progressively leading to impairments in the seminiferous epithelium and germ cells and alterations in semen parameters. Indeed, several studies have demonstrated that animals exposed to HH, whether in natural high-altitude environments or under simulated hypoxic conditions, exhibit damage to the self-renewal and differentiation of spermatogenesis, an increase in germline cell apoptosis, and structural alterations in the seminiferous tubules. One of the primary mechanisms associated with the inhibition of differentiation and an increase in apoptosis among germ cells is an elevated level of oxidative stress, which has been closely associated with HH exposure. Human studies have shown that individuals exposed to HH, such as mountaineers and alpinists, exhibit decreased sperm count, reduced motility, diminished viability, and increased sperm with abnormal morphology in their semen. This evidence strongly suggests that exposure to HH may be considered a significant risk factor that could elevate the prevalence of male infertility. This literature review aims to provide a comprehensive description and propose potential mechanisms that could elucidate the infertility processes induced by HH. By doing so, it contributes to expanding our understanding of the challenges posed by extreme environments on human physiology, opening new avenues for research in this field.

## 1. Introduction

Hypoxia is a condition characterized by insufficient O_2_ at different levels, resulting in several physiological manifestations. Different hypoxic types have been described, including hypobaric-hypoxia (HH) (high altitude), hypoxemic-hypoxia (low O_2_ tension in the arterial blood), anemic hypoxia (decrease in O_2_-carrying capacity), circulatory hypoxia (heart is unable to pump enough blood), and histotoxic hypoxia (unable to utilize O_2_ effectively) [1]. Among these, high-altitude HH stands out as one of the most inhospitable environments globally, primarily due to the reduction in barometric pressure, which triggers a complex array of physiological responses [2,3].

Humans subjected to HH experience several adaptative mechanisms, including heightened ventilation, a rise in hematocrit concentration and increased hemoglobin level, and precise regulation of pulmonary vasodilation and systemic vasoconstriction [4,5,6]. However, it is important to note that some individuals may experience acute mountain sickness, a clinical condition characterized by symptoms such as headache, vomiting, sleep disturbances, and desaturation. In more severe cases, exposure to high-altitude conditions can develop high-altitude pulmonary edema and high-altitude cerebral edema [7,8,9].

While high-altitude HH environments may be considered challenging, it is worth noting that more than 140 million people worldwide reside at altitudes above 2500 m [10,11]. In Western South America, vast mountain ranges, particularly the Andes, extend across the region [12]. The Andes Mountains stretch from the extreme north of Venezuela to the Chilean Patagonia, encompassing altitudes ranging from 2500 to 6000 m. At these heights, various human activities take place, including tourism, cross-border care services, education, and mining [13]. These geographically challenging conditions are marked by reduced barometric pressure (BP), diminished oxygen partial pressure (PO_2_), heightened ultraviolet (UV) radiation, and extreme temperatures, necessitating physiological adjustments in organisms to cope with these extreme environmental factors. Santoloya et al. and Gonzáles et al. [4,5] have indicated that indigenous communities inhabiting the Andean plateau exhibit moderate hyperventilation, increased hematocrit concentration, and elevated hemoglobin level as compensatory mechanisms for the lower ambient oxygen pressure, especially when compared to non-residents.

Mining activity is the economic mainstay for numerous countries, with many mining operations in high-altitude hypoxic environments. For instance, in countries like Chile, more than 38,000 individuals are exposed to altitudes exceeding 3000 m, experiencing intermittent hypobaric hypoxia (IHH) and/or intermittent chronic hypobaric hypoxia (ICHH) [14]. It is worth noting that, as per Chilean labor and health legislation, miners are not mandated to undergo altitude training. However, the legal framework does establish control measures, including health assessments for occupational exposure to high altitudes, occupational surveillance programs, and measures to mitigate the effects of hypobaric conditions [15]. Given that high-altitude environments lead to various physiological adaptations and that mining plays a crucial economic role in several countries, it becomes imperative to understand the consequences of HH exposure at multiple levels, including its effects on muscular function, autonomic responses, and reproduction. Such understanding can pave the way for innovative pharmacological and non-pharmacological strategies to counter the challenges posed by HH exposure.

The adaptation mechanisms in response to chronic HH (CHH) and CIHH often exhibit similarities, although their respective acclimatization timelines differ. Acclimatization to CHH is typically achieved within a few months, while adapting to CIHH requires a more extended period, ranging from 3 to 8 years. These adaptation mechanisms encompass preserving myocardial contractility, preventing apoptosis of cardiomyocytes, increasing coronary blood flow and promoting myocardial capillary angiogenesis, activating ATP-sensitive K^+^ channels, and inhibiting mitochondrial permeability transition pores [7]. Additionally, angiogenesis, a mechanism involved during exposure to HH, entails the creation of new blood vessels from pre-existing ones and is regulated by the vascular endothelial growth factor (VEGF). Hypoxia triggers the up-regulation of VEGF transcription through the hypoxia-inducible factor (HIF) pathway. This process enhances oxygen delivery to tissues in hypoxic conditions [8,9]. On the other hand, erythropoiesis, the process responsible for generating red blood cells, is orchestrated by the hormone erythropoietin (EPO). Hypoxia affects erythropoiesis by up-regulating EPO transcription via the HIF pathway, leading to an increase in red blood cell production. This augmentation contributes to an enhanced oxygen-carrying capacity of the blood [8,9].

In addition to the well-documented physiological compensations that occur in response to HH, research has also shown that HH can harm male fertility. This is associated with increased oxidative stress at the testicular level, leading to the deterioration of spermatogenesis and alterations in semen parameters [16,17,18,19]. The first observations of impaired human spermatogenesis associated with HH were made when a group of nine volunteers was exposed to an altitude of 4270 m for 45 days, decreasing sperm count [16]. Subsequent studies by Verratti et al. (2016) and Okamura et al. (2003) involving mountaineers exposed to high altitudes for 35 days reported declines in sperm count and a reduction in the percentage of spermatozoa with normal morphology [16,20]. Similar findings have been reported in pre-clinical models as well [21]. Additionally, a decrease in sperm count and reduced sperm normal morphology was observed in 10 mountaineers exposed to an altitude of 7821 m for 35 days [16,20]. Similar results have been previously observed in the rhesus monkey [21]. While the evidence regarding the negative impact of HH on male fertility is consistent, it is simultaneously limited by the lack of detailed explanations for the mechanisms underlying these effects. Therefore, the purpose of this manuscript is to provide a comprehensive review of the literature, summarizing the relevant findings regarding the effects of HH associated with high altitudes on spermatogenesis and semen parameters. Furthermore, we aim to describe and propose potential mechanisms to account for the damage to germ cells and the seminiferous epithelium induced by increased oxidative stress from HH exposure. 

## 2. Hypobaric Hypoxia

**Figure 1:**
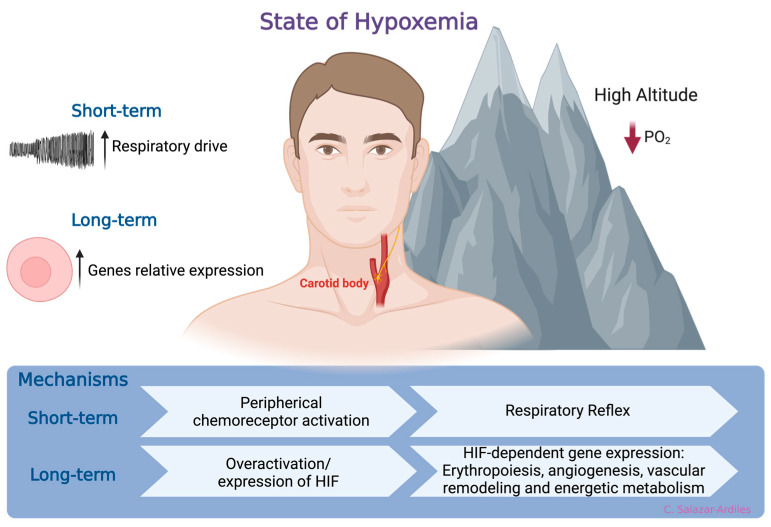
States of hypoxemia. When oxygen levels decrease, two distinct, time-related phenomena are triggered. In the short-term, a respiratory reflex is activated through peripheral chemoreceptors, specifically the carotid body. This reflex initiates a compensatory mechanism aimed at enhancing the availability and delivery of oxygen at the tissue level. Following more prolonged exposure to hypoxia, a molecular response is triggered. This response involves the hypoxia-inducible factor (HIF), which, in turn, induces gene expression related to processes such as erythropoiesis, angiogenesis, vascular remodeling, and energetic metabolism. Created with BioRender.com.

At sea level, the atmospheric oxygen (O_2_) pressure remains constant at 156 mmHg, constituting approximately 21% of the atmosphere. However, as geographical altitude increases, atmospheric pressure decreases, reducing the PO_2_. Consequently, this decreases the fraction of inspired O_2_, ultimately promoting a state of hypoxemia [22]. In response to hypoxemia due to exposure to HH, compensatory mechanisms have been identified that aim to enhance the availability and delivery of O_2_ at the tissue level [23]. The first mechanism is a respiratory reflex via peripheral chemoreceptor activation [24,25]. When HH exposure is prolonged, a molecular response is activated, which involves HIF, a member of a family of transcription factors. HIF induces gene expression related to erythropoiesis, angiogenesis, vascular remodeling, and energetic metabolism [26,27,28,29]. Activation of these mechanisms increases ventilatory response to hypoxia, enhancing oxygen uptake and triggering adaptive responses to short- and long-term high-altitude HH exposure [30] (Figure 1). 

In addition to the mechanisms mentioned earlier, exposure to HH results in an increase in oxidative stress. HH-induced oxidative stress, arising from an oxygen deficit, can lead to the accumulation of electrons. Without sufficient oxygen as the final electron acceptor, this electron buildup triggers superoxide anion formation (O_2_•). Subsequently, this superoxide anion can evolve into hydrogen peroxide (H_2_O_2_) and other reactive oxygen species (see Section 3) [7]. This cascade of reactive oxygen species (ROS) has been proposed as the underlying cause of various pathologies, including cardiovascular issues, neurodegenerative diseases, and male infertility [31]. Notably, it has been demonstrated that male fertility problems associated with HH are closely linked to oxidative stress at the testicular level [16,17,18,19]. However, the precise molecular mechanisms driving these physiological phenomena have not been comprehensively elucidated. It is essential to note that while there is evidence of compromised female fertility during exposure to HH, this aspect falls beyond the scope of the present manuscript [32].

## 3. Oxidative Stress 

Mitochondria serve as the central hub for adenosine triphosphate (ATP) production within the cell, primarily through processes like respiration and oxidative phosphorylation. However, this energy-generating mechanism has a notable consequence as it generates byproducts from highly reactive oxygen-derived molecules, known as ROS [33,34]. The primary ROS include superoxide anions (O_2_•), hydrogen peroxide (H_2_O_2_), peroxyl radicals (ROO•), and hydroxyl radicals (OH•) [35]. ROS play crucial roles at low levels as intermediaries in various cellular pathways. Nevertheless, when ROS levels surpass the cell’s antioxidant defense capacity, it can lead to oxidative stress, potentially causing cellular damage and dysfunction, including mitochondrial dysfunction [36,37,38].

Excessive production of O_2_• can damage mitochondrial DNA, causing mutations and deletions that compromise mitochondrial integrity and functionality. This primarily affects complexes I and IV of the electron transport chain, significantly reducing ATP production and impairing the cell ability to maintain ionic gradients. Consequently, greater ROS production occurs, negatively impacting cellular function and altering the structure of proteins, lipids, and DNA. If the cell intrinsic DNA repair mechanisms cannot restore the integrity of mitochondrial and nuclear DNA, the mitochondrial apoptosis signaling pathway is activated [34,39,40].

Several environmental factors, including HH, can induce an increase in ROS. HH involves a decrease in PO_2_, which reduces the oxygen supply to mitochondria, leading to an accumulation of electrons in the electron transport chain [7]. Under normal circumstances, O_2_ is the final electron acceptor in the mitochondrial electron transport chain [41]. However, in the presence of HH, the lack of oxygen as the final acceptor leads to the production of O_2_•. Subsequently, this anion can evolve into hydrogen peroxide (H_2_O_2_) and other ROS, generating an excess of ROS that triggers detrimental processes for the cell, such as lipid peroxidation, protein peroxidation, and DNA fragmentation [37].

Lipid peroxidation is a biochemical process in which lipids, such as fats and phospholipids, undergo damage due to their reaction with ROS or free radicals. This process is particularly relevant in plasma membranes, predominantly composed of polyunsaturated fatty acids, making them highly susceptible to ROS attack. The consequence of this reaction is the excessive production of malondialdehyde. This oxidation product adversely impacts the integrity and functioning of the cellular membrane and various cellular processes [31,42].

Proteins, like lipids, are highly susceptible to damage caused by ROS. The main target areas of ROS attack in proteins are the aromatic and heterocyclic rings of amino acid residues. The primary types of damage caused by ROS in proteins include the modification of amino acid residues, peptide bond breakage, alterations in the native three-dimensional structure of the protein, and the formation of cross-linked protein polymerization. These processes lead to disruptions in cellular protein components, ranging from misfolding of proteins to forming protein plaques and protein denaturation [43]. On the other hand, ROS-dependent DNA fragments can interact with DNA, causing lesions such as base oxidation and chain breaks. This oxidative damage can have significant consequences for health, as it affects DNA replication and transcription and compromises genome integrity [44,45].

**Figure 2:**
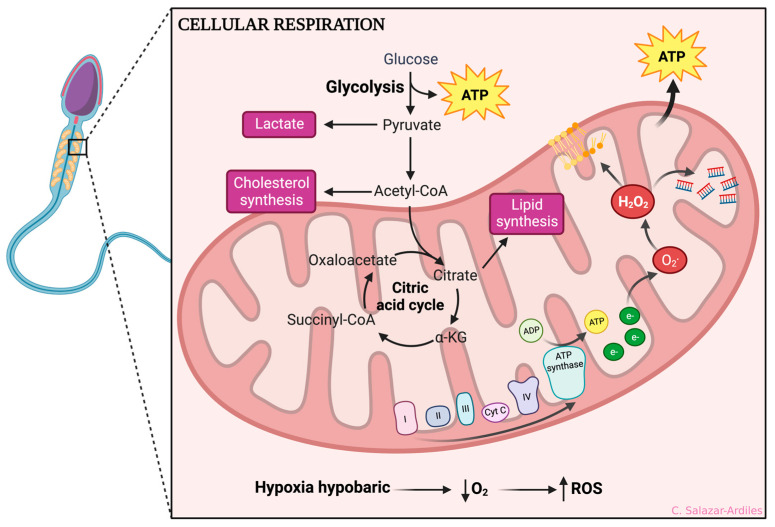
Hypoxic-dependent oxidative stress at the spermatozoa mitochondria. From glycolysis, ATP is obtained, and acetyl-CoA is formed, which enters the citric acid cycle. After that, oxidative phosphorylation is initiated to create ATP through different mitochondrial complexes. During HH, the complexes I and IV of the electron transport chain are affected by an accumulation of electrons, significantly reducing ATP production and impairing the cell ability to maintain ionic gradients. Consequently, ROS production occurs, including superoxide anions (O2•), hydrogen peroxide (H_2_O_2_), peroxyl radicals (ROO•), and hydroxyl radicals (OH•). ROS production negatively impacts cellular function, increasing lipid peroxidation, protein peroxidation, and DNA fragmentation. Created with BioRender.com.

The evidence shown so far makes it clear that sperm are highly sensitive to ROS production due to their limited antioxidant defense mechanisms and high content of polyunsaturated fatty acids. ROS can cause oxidative damage to sperm cell membranes, proteins, and DNA, leading to impaired motility, viability, and fertilization capacity [46] (Figure 2).

## 4. Morphology of the Seminiferous Tubules

Advancements in recent decades have enabled the development of 3D reconstruction techniques, providing detailed insights into the structure of the seminiferous tubules (STs) [47,48]. The STs form a complex network responsible for the daily production of millions of spermatozoa. They are organized into one to five hundred lobules, separated by connective tissue that further connects to the rete testis. Additionally, the efferent ductulus establishes communication with the head of the epididymis [49].

The seminiferous tubule is surrounded by (i) Peritubular myoid cells, flat cells that form smooth muscle layers around the STs, providing structural support and aiding contractions to move immotile sperm towards the rete testis; and (ii) Leydig cells, polygonal cell clusters within the interstitial tissue near blood vessels, responsible for testosterone production [50].

**Figure 3:**
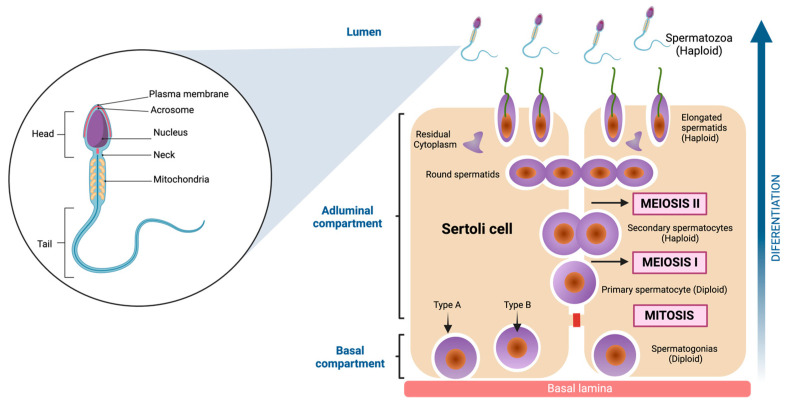
Spermatozoa structure and Spermatogenesis. On the left side of the figure, the main parts of the spermatozoa are shown, which are divided into three components (head, neck, and tail). To the right of the figure, the cell differentiation process is shown, which begins with the spermatogonia (diploid cell) and ends when spermatozoa (haploid cell) are obtained. The cells go through several stages, one mitosis (first stage) and two meiosis in the final stages. Created with BioRender.com.

The inner of the seminiferous tubules present a highly organized preestablished architecture, conformed by seminiferous epithelium, which is structured for two different types of cells. The first of these are Sertoli cells (SC), irregular columnar somatic cells attached to the basal lamina. They have a large nucleus with even chromatin distribution, surrounding and supporting germ cells. Tight junctions form the blood-testis barrier, dividing the seminiferous epithelium into basal and adluminal compartments [51]. The second are Germ cells. These cells comprise a family whose fundamental purpose is transforming into spermatozoa to transmit genetic information across generations. Morphological studies have allowed us to characterize the shape and structure of the germ cells [52,53]. Indeed, six types of germ cells have been identified in the adult testis, which differentiate into spermatozoa [54]. These include Spermatogonia, diploid, undifferentiated cells adjacent to the basement membrane, characterized by a dense nucleus housing a prominent nucleolus. Their extensive cytoplasm contains ribosomes, vesicular endoplasmic reticulum, and clustered mitochondria [52,55]. Primary Spermatocytes: Engaging in meiosis without direct basal lamina contact, these cells possess a well-developed Golgi apparatus and rough endoplasmic reticulum. Their nucleus assumes a spherical shape with filamentous chromatin, a characteristic of prophase I [56]. Secondary Spermatocytes: Haploid, ovoid cells with a nucleus containing prominent chromatin clumps. The cytoplasm is rich in rough endoplasmic reticulum [57]. Round Spermatids: These haploid cells exhibit a polygonal or spherical morphology, approximately 6 μm in diameter. Their small nucleus contains uniformly distributed chromatin. They undergo a transformative process to mature as spermatozoa [58]. Elongated spermatids: Elongated spermatids are highly polarized cells with a head that contains the genetic materials in highly condensed chromosomes on one end and a long tail constituted by actin- and microtubule (MT) -based cytoskeletal elements [59]. Spermatozoa: These highly diverse cells are recognized for mobility from the insemination site to fertilization. Variations in head shape and flagellum length are evident among different species. The acrosome, derived from the Golgi complex, stores enzymes essential for penetrating egg coats. The nucleus, occupying a significant portion of the head volume, is the repository for genetic information the male progenitor contributes. The flagellum is the mobile apparatus, segmented into the neck, middle piece, principal piece, and endpiece [60,61] (see Figure 3).

Several studies conducted in preclinical models have shown that the STs external and internal architecture is significantly damaged due to exposure to HH, attributed to ROS production, and subsequently, cell damage [62,63,64].

## 5. Hypoxia-Dependent Primary Structural Damage of the Seminiferous Tubules

Over the past decade, there has been a growing body of evidence derived from animal models, shedding light on the adverse impacts of exposure to hypoxia on the components of the STs. The initial in vivo studies that demonstrated the harmful effects of HH on spermatogenesis were conducted by Monge and Mori-Chávez (1924) and involved rabbits and cats exposed to 4400 m, revealing significant deterioration in the germinal epithelium [65]. Similarly, Saxena (1995), after exposure to a simulated altitude of 4411 m, reported damage to the germinal epithelium in Rhesus monkeys [21]. Moreover, Farias et al. (2005) showed that 60 days of simulated HH at 4600 m was able to promote a significant reduction in testicular mass, an increase in interstitial space, a decrease in the height of the seminiferous epithelium, and a depletion of cellular tissue [66]. Similar results were observed at high altitudes in the Chilean plateau (3260 m) [62].

Along with the previous observation, Liao et al. (2010), in rats exposed to 15 and 30 days at simulated high- altitude (5000 m), reported testicular abnormalities, such as germ cell degeneration, seminiferous epithelium disorganization, reduced tubule cellularity, and seminiferous epithelial vacuolation [67]. Furthermore, in the same manuscript, electronic microscopy revealed an augmented presence of lipid droplets within SCs, the formation of myelin-like structures in SCs cytoplasm, distended mitochondria characterized by the loss of cristae, degenerated spermatogonia featuring condensed and marginated chromatin, and sporadic nuclear envelope invaginations in primary spermatocytes [67]. 

In recent years, transcriptome research conducted in preclinical models has shed light on the effects of testicular exposure to HH. These investigations have revealed that such exposure leads to modifications in the expression of genes critical to the functioning of the seminiferous tubule, raising concerns about its potential negative impact on male fertility. Nevertheless, further research is imperative to comprehensively understand the implications of these alterations [68,69].

**Figure 4:**
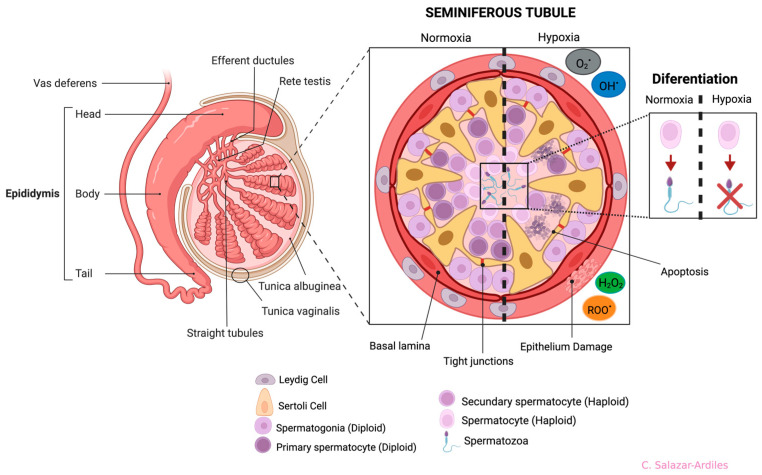
Anatomy of the testis and germline and effects of hypoxia on seminiferous tubule cells. Testis structures (left side): the epididymis is divided into four anatomical regions: the initial segment, head (caput), body (corpus), and tail (cauda). On the right side, a cross-section of the seminiferous tubule shows all cells participating in spermatogenesis. Cell differentiation is from the basal part of the tubule to the lumen, starting from spermatogonia to spermatozoon. The seminiferous tubule is divided in two (dotted line), showing normoxia (left) and hypoxia (right). During hypoxia, it shows cell abnormalities ranging from cell apoptosis and damage to the epithelium, triggering differentiation problems. Each cell type, including the Sertoli and Leydig Cells, is shown at the bottom. Created with BioRender.com.

The most frequently described damages resulting from exposure to both short and long-term HH are alterations in the organization of the STs. These alterations include a reduction in the number of SCs, an increase in the diameter of the seminiferous tubule, and the folding of the basement membrane [63,66,70,71] (Figure 4).

## 6. Principal Damages Produced to Spermatogenesis by Hypoxia 

Spermatogenesis is a complex process of self-renew and differentiation of the germinal diploid cells culminating with the production of mature spermatozoa [72,73,74] (see Figure 3). A-dark spermatogonia enter mitosis, and one daughter cell remains spermatogonia-Ad, while the other acquires A-pale properties (Ap). Ap spermatogonia enters mitosis, and the daughter cells are then named B-spermatogonia. Then, B-spermatogonia actively proliferate and enter meiosis, thus named pre-Leptotene primary spermatocytes. Meiosis proceeds through the Pre-Lep, leptotene, zygotene, and pachytene stages, where DNA replicates, chromosomes with chromatids pair with their homologous chromosomes, and chromatids suffer meiotic recombination. After this, pachytene primary spermatocytes suffer the first meiotic division, with the separation of homologous chromosomes, resulting in haploid secondary spermatocytes. The STs cells immediately suffer the second meiotic division, with the separation of the chromatid. The resultant haploid round spermatids enter a morphological, structural, and physiological differentiation process named spermiogenesis. During spermiogenesis, the flagellum is extruded, the nucleus condenses and elongates, and the acrosomal vesicle covers the upper nuclear region [75,76]. Since spermatogenesis is highly regulated, extreme environments such as HH are capable of altering it. Indeed, evidence shows that HH generates random apoptosis and inhibition of differentiation and self-renewal throughout the germ line [67,71]. 

It has been estimated that an adult human being produces approximately 45 million sperm per day, with an average velocity of around 1000 sperm/s [72]. To sustain this high rate of sperm production, a population of stem cells with substantial proliferative capacity and continued oxygen availability for ATP production is required to support self-renewal and the differentiation of spermatogonia [77]. Previous research has revealed that PO_2_ in the seminiferous epithelium is significantly low, measuring approximately 2 mmHg. This low oxygen level is likely associated with the distance that oxygen must traverse through the tissue and the substantial oxygen consumption by the spermatogonia [78,79]. Consequently, spermatogonia stem cells predominantly rely on glycolysis as their primary metabolic pathway for ATP production. This metabolic strategy sustains their self-renewal and helps safeguard their cellular components from oxidative stress-induced damage [80,81,82]. Nonetheless, spermatogonia must be adaptable in their metabolic processes, shifting between glycolysis and oxidative phosphorylation to ensure a sufficient supply of energy (ATP) that can support the proliferation and differentiation of germ cells, all while considering the availability of oxygen [78,83]. Chen et al. demonstrated that oxidative stress resulting from oxidative phosphorylation can induce gene expression that promotes spermatogonia differentiation. Furthermore, they reported that inhibiting both glycolysis and oxidative phosphorylation leads to a reduction in the number of spermatogonia expressing signaling molecules associated with self-renewal and differentiation [84]. Therefore, it is plausible that exposure to conditions that induce oxidative stress, such as HH, could potentially disrupt the differentiation of spermatogonia.

Studies conducted on rats exposed to HH have demonstrated a loss of germ cells in all cell cycle stages. González et al. [85] and Farias et al. [86], in studies involving rats exposed to simulated high-altitude hypoxia at 4200 m, reported a reduction in the germ cell population, particularly spermatogonia and spermatocytes. Additionally, in a study conducted by Bai et al. [71] on rats exposed to simulated high-altitude hypoxia at 3000 m, a significant increase in the apoptosis rate of germ cells, particularly spermatogonia and spermatocytes, was reported. Additionally, it has been observed that exposure to HH leads to significant changes in testicular vascularization, increasing intratesticular temperature. This increase in temperature triggers the inhibition of germ cell proliferation and leads to the arrest of spermatogenesis [87].

The reduction in the population of germ cells has been attributed to an increase in the rate of apoptosis associated with oxidative stress in spermatogonia and spermatocytes [71]. In support of this notion, Liu et al. [79] demonstrated that exposure to HH increases the apoptosis of round and elongated spermatids. It is important to note that apoptosis can be triggered by various stimuli, resulting in physiological alterations at the endocrine level [88]. One such physiological mechanism linked to apoptosis is associated with the hypothalamus, pituitary gland, and testis axis. It has been observed that exposure to HH leads to a decrease in the content of gonadotropin-releasing hormone (GnRH) in hypothalamic neurons. This decrease in GnRH subsequently reduces the synthesis of follicle-stimulating hormone (FSH) and luteinizing hormone (LH). These changes affect the steroidogenesis in Leydig cells, resulting in a reduction in the concentration of plasma testosterone. This reduction in testosterone, in turn, contributes to an increase in the apoptosis rate among germ cells [89,90,91].

The evidence mentioned above indicates that exposure to HH inhibits spermatogonia and spermatocyte differentiation, increases the rate of germ cell apoptosis, and alters the structural conformation of the seminiferous tubule (Figure 4).

## 7. Semen Parameters and Hypobaric Hypoxia 

The male reproductive capacity depends mainly on the functionality and structural integrity of its spermatozoa [92,93,94]. The World Health Organization (WHO) establishes that the quality of semen can be measured by assessing the following sperm parameters: ejaculated volume (1.4 mL), total sperm count (>39 × 10^6^/ejaculated), total motility (42%), progressive motility (30%), morphology (4% normal sperm), and viable sperm (54%) [95]. Although the evidence is limited, it has been shown that exposure to HH can promote negative consequences in these semen parameters [62,79,85,86]. 

Saxena [21], using Rhesus monkeys exposed to HH for twenty-one days, simulating an altitude of 4411 m, showed a significant reduction in semen volume, sperm concentration, and motility. In addition, two studies have shown that rats exposed to HH increase the percentage of fragmentation of the sperm DNA [71,79]. From a human perspective, Okumura et al. [16], in three healthy men exposed to HH for 90 days at 5100 m, showed through semen analyses that there was a decreased sperm concentration (−21%) and an increase in the percentage of sperm with abnormal morphology (35%). Similarly, a significant reduction in the quality of the semen parameters (concentration, motility, viability, and morphology) has been observed in trekkers, mountaineers, tourists, and workers [17,18,20]. Semen analysis of subjects exposed to 3600 m for one month showed several types of sperm structure abnormalities, such as spermatozoa with small or large heads, double or multiple heads, headless, crimp neck, expansion neck, tailless, double tail, short tail, and coiled tail [19].

The evidence strongly indicates that sperm are highly sensitive to ROS due to their limited antioxidant defense mechanisms and high content of polyunsaturated fatty acids. ROS can lead to oxidative damage in sperm cell membranes, proteins, and DNA, ultimately reducing motility, viability, and fertilizing capacity (for more details, see Section 3) [96,97]. Studies have demonstrated that the decrease in sperm concentration is associated with an increase in oxidative stress at the testicular level due to hypoxia. This oxidative stress can cause fragmentation in the nuclear DNA structure of germ cells, as indicated by the presence of 8-hydroxy-2’-deoxyguanosine during exposure to HH. Damage to nuclear DNA can trigger either the mitochondrial apoptotic signaling pathway or death receptors, leading to an increased apoptosis rate, primarily affecting spermatogonia and primary spermatocytes. Oxidative stress is a crucial factor contributing to reducing sperm concentration [67,98,99,100]. Additionally, an imbalance in the metabolic pathways, specifically the shift between glycolysis and oxidative phosphorylation due to increased oxidative stress, may inhibit gene expression necessary for the self-renewal of spermatogonia and their differentiation into primary spermatocytes. This metabolic disturbance could also explain the reduction in sperm concentration following exposure to HH [84]. In fact, in a study conducted by Saxena, a significant increase in glucose levels in the semen fluid of Rhesus monkeys was documented [21].

In addition to the previously mentioned factors, hypobaric hypoxia (HH)-induced oxidative stress can damage DNA, such as nitrogenous base deletions, mutations, and polymorphisms. This damage may result in a failed exchange of histones to protamines, leading to lower sperm condensation or packing [101,102,103], provoking epigenetic regulation [104]. Furthermore, exposure to HH has been shown to alter the pattern of histone methylation at the N-terminal ends and induce changes in the process of histone-to-protamine exchange during spermiogenesis. These alterations can lead to lower compaction of sperm DNA. These two mechanisms, DNA damage and histone-to-protamine exchange, primarily contribute to an increase in the percentage of sperm with abnormal head morphology.

According to the WHO Manual for Analysis of Human Semen and Sperm Interaction with Cervical Mucus, sperm motility is one of the essential parameters, given that the motility allows sperm to migrate from the insemination site to the fertilization area [95]. Of note, it has been shown that oxidative stress also promotes the deterioration of sperm motility [71,79,105]. Indeed, low synthesis of ATP is proposed as one of the essential mechanisms related to the loss of sperm motility during HH due to a metabolic shift [106]. Luo et al. observed that, in human sperm, HH promotes an increment of mitochondrial DNA copies, which has been proposed as a compensation mechanism for low ATP production [107]. Another study shows that a high degree of oxidative stress in the semen causes a decreased activity in the enzyme glucose-6-phosphate dehydrogenase, contributing to reduced sperm motility due to a metabolic deficiency [108].

Peroxidation of unsaturated fatty acids by increased oxidative stress has been proposed as another mechanism that contributes to loss of motility in sperm, altering the fluidity and, therefore, leading to a decrease in the motility of the sperm flagellum [105]. Besides, it has been described that excessive oxidative stress causes protein oxidation that leads to the crisscrossing of the peptide chains and the formation of carbonyl groups, which damages the integrity of the structural protein [109,110]. Indeed, this allows hypothesizing that the increase in oxidative stress induced by exposure to HH can generate damage to the structural protein that makes up the axoneme, dysplasia of the outer dense fibers, and the fibrous sheath.

## 8. Conclusions

The present review depicts that from pre-clinical models to human beings, the spermatogenic process is quite susceptible to oxidative stress prompted by HH, which induces a rise in the apoptotic rate of germ line cells, inhibiting the differentiation of the spermatogonia to primary spermatocytes. Furthermore, an increase in oxidative stress at the testicular level and semen produces damage to the sperm structure, which is related to a reduction in the quality of the semen parameters, suggesting that exposure to HH could be a risk factor that increases the prevalence of male infertility. Nevertheless, it is necessary to obtain more evidence to establish a relationship between exposure to HH and male infertility.

## Data Availability

Data sharing not applicable.
